# Influence of In Vitro Digestion on Composition, Bioaccessibility and Antioxidant Activity of Food Polyphenols—A Non-Systematic Review

**DOI:** 10.3390/nu12051401

**Published:** 2020-05-13

**Authors:** Karolina Wojtunik-Kulesza, Anna Oniszczuk, Tomasz Oniszczuk, Maciej Combrzyński, Dominika Nowakowska, Arkadiusz Matwijczuk

**Affiliations:** 1 Department of Inorganic Chemistry, Medical University of Lublin, Chodźki 4a, 20-093 Lublin, Poland; k.wojtunik@o2.pl; 2 Department of Thermal Technology and Food Process Engineering, University of Life Sciences in Lublin, Głęboka 31, 20-612 Lublin, Poland; 3 Department of General Ophthalmology, Medical University of Lublin, Chmielna 1, 20-079 Lublin, Poland; dominika.nowakowska@umlub.pl; 4 Department of Physics, University of Life Sciences in Lublin, Akademicka 13, 20-950 Lublin, Poland

**Keywords:** plant metabolites, polyphenols, antioxidants, gastrointenstinal digestion, bioaccessibility

## Abstract

There is increased interest in following a healthy lifestyle and consuming a substantial portion of secondary plant metabolites, such as polyphenols, due to their benefits for the human body. Food products enriched with various forms of fruits and vegetables are sources of pro-health components. Nevertheless, in many cases, the level of their activities is changed in in vivo conditions. The changes are strictly connected with processes in the digestive system that transfigure the structure of the active compounds and simultaneously keep or modify their biological activities. Much attention has focused on their bioavailability, a prerequisite for further physiological functions. As human studies are time consuming, costly and restricted by ethical concerns, in vitro models for investigating the effects of digestion on these compounds have been developed to predict their release from the food matrix, as well as their bioaccessibility. Most typically, models simulate digestion in the oral cavity, the stomach, the small intestine and, occasionally, the large intestine. The presented review aims to discuss the impact of in vitro digestion on the composition, bioaccessibility and antioxidant activity of food polyphenols. Additionally, we consider the influence of pH on antioxidant changes in the aforementioned substances.

## 1. Introduction

*‘Let food be thy medicine, and medicine be thy food.’* Hippocrates’ famous quote conveys the true role of food in our lives. Our organism, as a whole and in its constituent parts, requires nourishment, the source of components and energy necessary for the continuation of existence. It is obvious that cells cannot change their position in the body and travel to a food source. This fact brings about the situation that the food must be transported to the cells in an appropriate usable form that can be easily absorbed by the body structures. The digestive system, being a complex mechanism of mechanical and chemical transitions, provides the body with water, electrolytes and nutrients in the aforementioned bioavailable forms. 

In order to explain and understand the digestive mechanism, definitions of bioavailability and bioaccessibility are crucial. Bioavailability is a wide-ranging issue which includes gastrointestinal digestion, absorption, metabolism, tissue distribution and bioactivity. With reference to nutrition, bioavailability refers to the fraction of the nutrient that is stored or is available in physiological functions. It is a key term for nutritional effectiveness, as not all the amounts of bioactive compounds are used effectively by the organism. In other words, bioavailability expresses the fraction of ingested nutrient or bioactive compound that reaches the systemic circulation and is ultimately utilized. The term includes bioaccessibility, which is defined as the quantity of a compound that is released from its matrix in the gastroinstestinal tract, becoming available for absorption. The term includes digestive transformations of foods into material ready for assimilation, the absorption/assimilation into intestinal qpithelium cells as well as the presystemic, intestinal and hepatic metabolism [1].

Each part of the system is responsible for various food transitions, leading to the simplest particles being made available to the body’s cells. Further, well-known components of proteins, carbohydrates and fats, active components such as secondary plant metabolites are of significance in a healthy body [2]. Constituents such as these do not play building or energy-supply roles, but provide equally important cell protection, among which the antioxidant role is among the most important [3]. 

Nowadays, increasing numbers of people pay attention to following healthy lifestyles—of which, appropriate food intake is a key issue. They understand the need for the intake of pro-health components such as the aforementioned antioxidants, fiber, etc. In addition to fruits and vegetables rich in natural vitamins, antioxidants and minerals, functional food products constitute interesting parts of general food intake. Food products, enriched with various forms of fruits and vegetables, are bona fide sources of pro-health components. Among these, polyphenols, along with flavonoids, play pivotal roles. Secondary plant metabolites express numerous biological activities such as antiinflammatory, antioxidant, antibacterial, glucose level alignment, etc., [4,5,6]. The activities of secondary plant metabolites in the digestive system are various. It is known that biological activity is significantly different in in vitro and in vivo conditions. The changes are strictly connected with processes within the digestive system that transmute the structure of the active compounds and simultaneously keep or modify their biological activities. This is the subject of our scientific consideration.

The presented review aims to discuss the impact of in vitro digestion on the composition, bioaccessibility, and antioxidant activity of food polyphenols. Additionally, we will consider structure–antioxidant activity relationships and the influence of pH on antioxidant changes in the aforementioned substances.

## 2. Basis of Digestive System Mechanism

Preserving human health and well-being is closely related to the digestion of food, which is source of energy and necessary components, macro- and microelements. The form of the nutritional components released during digestion depends on both initial food properties as well as transformations during the digestive mechanistic and chemical processes [7].

In general, digestion can be described as a complex process in which nutrients from food are subject to processes which lead to using them for energy, growth and cell repair. The processes take place in the digestive tract, which can be described as a long twisting tube that starts at the mouth and ends at the anus. The tract can be divided into several parts—each of which is necessary for proper digestion—including the mouth, esophagus, stomach, small intestine, large intestine and anus [8]. In addition to the aforementioned parts, accessory organs, such as salivary glands, liver, pancreas and gall bladder play indispensable roles in the digestion process in which proteins are broken down into amino acids, fats are reduced to fatty acids and glycerol or carbohydrates devolve into simple sugars, while the remaining molecules (e.g., secondary plant metabolites), depending on structure and chemical character, are transformed into other molecules. It is known that proper digestive functioning requires an appropriate pH as well as the activity of healthy microbiota. The part and the associated digestive process are listed in Table 1. 

Aside from the most important nutrients such as peptides, carbohydrates and lipids that act as energy and cell building sources, digested food also includes other types of substances that are responsible for cell protection and regeneration. Among them are secondary plant metabolites (e.g., polyphenols, terpenes) various in terms of their biological activity. The activity as well as effective cellular absorption is strictly related with their chemical structure. In most cases, the biological activity of the compounds depends on the digestion condition, namely pH and enzyme availability. Current data suggest that the biological activity of polyphenols, the most studied secondary metabolites, is closely related to the pH of the experiment environment (in vitro and in vivo studies) [11,19,20]. This issue is analyzed below.

## 3. Digestion Models for Testing the Bioaccessibility of Secondary Plant Metabolites

A serious problem for the interpretation of phytochemical bioaccessibility based on in vitro studies is the diversity of models. This hampers a comparison of results across all studies. The utilized models mainly differ in the incorporation of various digestion stages, pH, digestion times, the nature of digestive enzymes and concentrations of electrolytes and bile acids. In addition, most of the models function in the static mode. While there are dynamic models that mimic the continuous changes in the physicochemical conditions, these models are rare and much more labor and cost intensive than the static models [21].

### 3.1. Static Models

The simulation of the digestive process can be divided into two major stages: simulating gastric and small intestinal digestions. Adaptations to this model have been made to modify the conditions and the procedures for studies of the digestibility and bioaccessibility of phytochemicals, but the physiological conditions chosen vary considerably across different static in vitro studies [21]. 

Static modeling of the gastric digestion of phytochemicals is basically conducted by pepsin hydrolysis of homogenized samples under fixed pH and temperature for a period of time. The internal body temperature (37 °C) is generally used. Dynamic processes occurring during human digestion such as mechanical forces or continuous changes in pH and secretion flow rates are usually not reproduced. The major differences among the methods used for modeling the gastric phase of digestion are the addition or absence of phospholipid vesicles, the addition or absence of lipase, an incubation time between 0.5 and 2 h, pH varying from 1.7 to 2.5 and pepsin to substrate ratio [21]. 

Static models are particularly useful where there is limited digestion (e.g., gastric or intestinal steps), but are less applicable in total digestion studies. These methods can be used to evaluate the influence of digestion conditions and to carry out studies on the effect of food structure and composition, dietetic factors and food processing on nutrient and bioactive compound bioaccessibility in order to establish the nutritional value of foods and improve food design. Static models are predominantly used for digestion studies on simple foods and isolated or purified food components [22].

Various static digestion models have been proposed, which often impedes the possibility of comparing results across research teams. For example, a large variety of enzymes from different sources has been used and these enzymes differ in their activity and characterization. Differences in pH, mineral composition and digestion time that alter enzyme activity and other phenomena may also significantly alter results [22]. Several studies have investigated the flow pattern of systems such as the Dissolution Apparatus USP at various speeds by using Computational Fluid Dynamics. However, the hydrodynamics of these systems are far from that calculated for the human stomach. In fact, specimen dissolution is greatly influenced by fluid flow and mechanical forces, and this must be taken into account when designing an in vitro method which aims to predict the in vivo behavior of a formulation. Thus, a more comprehensive simulation of gastric digestion should not only mimic the biochemistry of the process but also its mechanical forces, since only a combined approach of the two will result in a close simulation of the in vivo scenario [23].

An important aim of the scientist was to standardize and to harmonize in vitro digestion while keeping the method sufficiently simple to reproduce all over the world. The static protocol for simulating digestion in the upper gastrointestinal tract published by INFOGEST was the result of more than 2 years of work involving extensive discussion among scientists from a wide range of relevant disciplines. INFOGEST is an in vitro static digestion method that uses constant ratios of meal to digestive fluids and a constant pH for each step of the digestion process. This makes the method simple to use but not suitable for simulating digestion kinetics. Using this method, food samples are subjected to sequential oral, gastric and intestinal digestion, while parameters such as electrolytes, enzymes, bile, dilution, pH and time of digestion are based on available physiological data. The clear definition of standardized experimental conditions and procedures is among the major advantages of the INFOGEST method. 

However, static digestion methods have known limitations and cannot mimic the complex dynamics of the digestion process or the physiological interactions with the host. For example, for the gastric phase, the pH is kept constant, and there is a lack of the gradual addition of gastric fluid and an absence of gradual gastric emptying. In addition, the enzyme activity in each digestive phase is kept constant, regardless of the type of food and whether the food contains high or low amounts of proteins, lipids, and carbohydrates. The intestinal phase is treated as one phase rather than as sequential duodenal, jejunal and ileal phases, which exhibit different dilutions, mineral content, pH, enzyme activities and microbial content [22,24,25,26]. 

In some cases, a slight alteration of the procedure can be considered to more accurately reflect physiological conditions. The new, amended and improved digestion method, INFOGEST 2.0, avoids challenges associated with the original method, such as the inclusion of the oral phase and the use of gastric lipase. The method can be used to assess the endpoints resulting from the digestion of foods by analyzing the digestion products and evaluating the release of micronutrients from the food matrix. In the study of the bioaccessibility of phytochemicals such as polyphenols and carotenoids, the model allows realistic release from a food into the aqueous phase. However, specific hydrolytic processes occurring at the brush border are currently not simulated, and additional steps, such as centrifugation of the digesta, are needed to separate the bioaccessible phases. An extension including colonic fermentation, an important step in the bioactivation of several phytochemicals, would further enhance the physiological appropriateness [22,24].

Many adaptations of the static model have been carried out for the investigation of various compounds, such as ultracentrifugation and/or filtration, to study the micellar phase of lipophilic constituents. For polyphenol bioaccessibility, additional steps such as dialysis have occasionally been introduced [21,27]. Unfortunately, static models cannot take into account dynamic physiological responses to the introduction of a food bolus, such as the pH increase and the following pH decrease in the stomach, and enzyme secretions in response to the food bolus introduced [21].

### 3.2. Dynamic Models

Different dynamic gastric models have been developed and designed for detailed measurement of gastric biochemistry and mixing. Dynamic models can simulate the continuous changes in the physicochemical conditions, including variation of pH from the mouth to the stomach and the intestine, altering enzyme secretion concentrations, and peristaltic forces in the gastrointestinal tract.

The dynamic gastric model (DGM) that was developed by the UK Institute of Food Research allows control in the stomach, among other parameters, over acidity, the quantity and rate of digestive enzyme release and the physical mixing of stomach contents. 

DGM is composed of two successive compartments [27]. The model reproduces gastric emptying and secretion according to data derived from echoplanar magnetic resonance imaging and the rates of gastrointestinal digestion obtained from human studies [28].

Different dynamic gastric models such as TNO-Intestinal Models (TIMs) have been developed by the Netherlands Organization for Applied Scientific Research. This computer-backed system allows studies to be performed on nutrient absorption, interactions between nutritional and functional food compounds, how food processing affects the nutritional qualities of food and the effectiveness of prebiotics throughout the digestive tract. The system is available in two complementary parts [29]. TIM-1 consists of four different compartments, representing the stomach, duodenal, jejunal and ileal parts of the gastrointestinal tract. TIM-1 enables the simulation of the gastric emptying rate, peristaltic movements and transit time through the small intestine, as well as gradual pH changes in the different compartments [30,31,32]. The limitations of the TIM systems include that there is no intestinal mucosa, and therefore absorption should be studied in combination with intestinal cell lines or tissues, and that the availability for absorption (bioaccessibility) is measured rather than the bioavailability including metabolism and excretion—this can be overcome by combining the TIM system with in silico modelling [33]. Both static and dynamic models that do not take into account the simulation of the colon have limitations in predicting the bioavailability of polyphenols. However, with the development of additional models aiming to simulate colonic fermentation, such as TIM-2, the non-bioaccessible fraction following gastric and small intestinal digestion may be studied [34]. In response to demand, a TIM system (tiny-TIM) has been designed. This tiny-TIM system closely mimics the events in the lumen of stomach and small intestine in an accurately controlled way. Tiny-TIM is a simplified version of TIM-1, designed to increase the throughput as compared to TIM-1, with a focus on studies that do not need separate intestinal steps. The tiny-TIM is used with the same gastric compartment as TIM-1. In the standard gastric compartment, the meal is mixed to obtain a homogenized gastric content and a consequent predictable gastric emptying of compounds. This is particularly important in order to compare the digestion of compounds under exactly controlled conditions. In order to include the effect of gastric motility on the gastric behavior of food components and pharmaceuticals, a gastric compartment is designed that mimics the shape and motility of the stomach in a more realistic manner. The new TIM Advanced Gastric Compartment (TIM-agc) system consists of a part with a flexible wall that gradually contracts to simulate gastric tone and consequent reduction in gastric volume during emptying. Two antral units can be moved to simulate mixing by an antral wave. Similar to other TIM models, the contractions are achieved by modulating the pressure on water that is circulated in the space between a glass jacket and a flexible membrane. All contractile movements and the resulting mixing and pressure profiles are accurately controlled and synchronized [35].

The DIDGI^®^ system was conceived in order to monitor the disintegration and the kinetics of the hydrolysis of the food occurring during a simulated digestion. The system focuses on the stomach and the small intestine. To be physiologically realistic, the system reproduces the gastric and intestinal transit times, the kinetics of gastric and intestinal pH, the sequential addition of digestive secretions and the stirring of the stomach and small intestine contents. The DIDGI^®^ system consists of two consecutive compartments simulating the stomach and the small intestine. The system is equipped with temperature, pH and redox sensors and variable speed pumps to control the flow of meal, HCl, NaHCO_3_, bile, enzymes and the emptying of each compartment. Flow rates are regulated by specific computer-controlled peristaltic pumps. Anaerobic conditions can be simulated by purging air with nitrogen. A Teflon membrane with a pore diameter of 2 mm is placed before the transfer pump, between the gastric and the intestinal compartment, to mimic the sieving effect of the pylorus in humans. The main advantage of this system is that it can handle real foods and full meals up to 200 g. Biochemical and physical processes are well mimicked. In contrast, mixing in the compartments only consists of basic stirring using a propeller and peristalsis occurring in the stomach, which is is not realistic in this system. So far, another limit is the absence of nutrient absorption in the small intestine due to the lack of dialysis membranes in the intestinal compartment. This limitation is currently being overcome by the development of a new version of the DIDGI^®^ [33].

Another example is the human gastric simulator (HGS), a model developed at the University of California-Davis. The HGS is composed of a latex chamber surrounded by a mechanical driving system to effectively mimic the frequency and intensity of the peristaltic movements in the stomach. HGS is designed to mimic the gastric shear forces and stomach grinding. This appears to be important for bioaccessibility studies, as the rate of release of phytochemicals, from fibrous particles, into the surrounding intestinal fluid is inversely proportional to particle size and directly proportional to phytochemical gradient. It is furthermore affected by the physical state of the phytochemical, the physical structure, and the surface properties of the particle [34]. 

Summarizing, static models provide an inexpensive means to assess multiple experimental conditions, allowing large numbers of samples to be tested. Dynamic multistage continuous models facilitate long-term studies and come closest to in vivo conditions. These complex computer-controlled systems, however, are expensive, more labor intensive and time consuming, and require higher operating costs [34].

### 3.3. Colonic Models 

The variety of in vitro colonic models is diverse, ranging from batch fecal incubations using a strictly anaerobic and dense fecal microbiota suitable for metabolic studies [22,35] to more complex continuous models involving one or multiple connected, pH-controlled vessels to mimic different parts of the human colon [36] or in vitro dynamic gastrointestinal–colonic system models [37]. The limitations of in vitro colonic models include that they may not fully represent the microbiota present in the colonic lumen and mucosa and that the combined rates of catabolism and absorption that occur in vivo are not reproduced. Static or batch models are of particular interest for a 1st assessment of colonic metabolism of phenolic compounds, which can be complicated by a high interindividual variability, and are used for comparison of different sources or doses of compounds. Dynamic, multicompartment colonic models are useful for long-term experiments needed to evaluate the spatial and temporal adaptation of the colonic microbiota to dietary phenolic compounds and the microbial metabolism of these phytochemicals. These models are designed to and should harbor a reproducible microbial community that should be stable upon inoculation, colon region specific, and relevant to in vivo conditions. The simulation of intestinal absorption to remove end products of microbial metabolism is also relevant to prevent inhibition of the colonic microbiota during in vitro studies [34]. Regrettably, the capacity of colonic models to simulate the in vivo conditions is limited by the lack of studies involving the formation of microbial biofilms adhering to the colonic epithelium.

ARCOL (for ‘ARtificial COLon’) is a one-stage fermentation model that reproduces the colonic environment of humans or animals. It is the first model that has allowed the maintaining of anaerobiosis inside the fermentor by the sole metabolic activity of the microbiota and not by flushing with N_2_ or CO_2_. The system integrates the main parameters of in vivo fermentation in the large intestine, such as pH, temperature, anaerobiosis, supply of simulated ileal effluents, colonic residence time, presence of a complex, high-density, metabolically-active microbiota and passive absorption of water and microbial metabolites.

Among available colonic in vitro models, ARCOL is among the few wireless systems that allow the maintenance of anaerobic conditions by the unique activity of intestinal microbiota and which are equipped with dialysis fibers in order to mimic passive absorption of microbial products. The effect of the single or repeated administration of compounds of interest on intestinal microbiota composition and activity can be evaluated in the ARCOL model [33].

## 4. In Vitro Digestion Stages 

### 4.1. Mouth Stage

In the mouth, food is subject to numerous chemical, biochemical and mechanical processes. Components of food may undergo the following changes: pH, ionic strength, temperature, action of various digestive enzymes (notably lingual lipase, amylase, protease); interactions with biopolymers in the saliva (mucin); interactions with sensory receptors of the tongue and mouth; and particle size reduction in bolus by chewing. These are important factors to take into consideration when designing an in vitro digestion step [38]. Mixing of simulated saliva and the introduced food bolus is desired, typically in a ratio of 1:1 [39]. Most often, in vitro methods are initiated using α-amylase at pH 7. Usually, an oral digestion phase is recommended only for foods rich in carbohydrates, due to the short interaction of oral enzymes with the food. Alternatively, research that starts with particles of a small size (50 to 1000 μm) may be appropriate, as these mimic the particle size following the chewing process for vegetables and fruits [40]. If oral digestion is omitted, dry samples may be introduced at a ratio of approximately 1:4 (food: liquid), considering common meal sizes of approximately 200 to 300 g and a gastric juice volume of approximately 1 L [41].

### 4.2. Gastric Stage

Reliable information on the breakdown of food constituents in stomach is crucial for assessing the bioaccessibility of phytochemicals for both static and dynamic methods. This digestion stage is a complex process that includes mechanical actions and the activity of gastric fluids. Gastric juice contains hydrochloric acid, pepsinogens, lipase, mucus, electrolytes and water. The rate of secretion of gastric juice varies from approximately 1 to 4 mL/min under fasting conditions to between 1 and 10 mL/min after food intake. The content of hydrochloric acid contributes to the denaturation of proteins and it activates pepsin. Peristaltic waves originating from the stomach participate in reducing the size of solid foods down to a diameter of 1 to 2 mm. Stomach emptying is a critical step in the digestion process. Several factors may influence the gastric emptying of food and fluids including volume, viscosity and pH. The duration depends on the physical properties and amounts to 3 to 4 h [42]. The gastric pH in the fasted state in healthy human subjects is in the range of 1.3 to 2.5. The intake of a meal generally increases the pH to above 4.5 depending on the buffering capacity of the food. Most static in vitro studies have been conducted at a pH below 2.5, which is a pH related to the human fasting state rather than to real food digestion, and the change in gastric pH is taken into consideration only in dynamic models [43]. Pepsin has been integrated in most in vitro models of gastric digestion, although in varying amounts, and pepsin content should be assessed as enzymatic activity per weight of protein for the sake of comparison. In contrast, gastric lipase is usually omitted in most in vitro models. This is not a good solution, because lipid digestion starts in the stomach with the action of preduodenal lipase on triacylglycerides and some other esters. Most of the lipids from diet are present as emulsified droplets, with diameters in the range of 20 to 40 μm, and it was suggested that gastric lipolysis can help to increase emulsification in the stomach, which would thus enhance lipophilic phytochemical bioaccessibility. Gastric lipolysis not only contributes to the overall digestion of triacylglycerides but it also triggers the subsequent action of pancreatic lipase on lipid substrates that may be poorly digested by pancreatic lipase alone; examples include milk fat droplets and lecithin-stabilized triacylglyceride emulsions. It is therefore recommended to add gastric lipase during the gastric phase of in vitro digestion [24].

### 4.3. Small Intestinal Stage

The in vitro small intestinal digestion of food involves mimicking pH, temperature, time and pancreatic juice including electrolytes, bile salts and enzymes. In the fed state, pH can vary from 5.4 to 7.5 in the duodenum [44], to 5.3 to 8.1 in the jejunum, and up to 7.0 to 7.5 in the ileum. Pancreatic enzymes, including proteases, amylases and lipases, act together with other digestive enzymes (maltase, lactase, α-dextrinase, and peptidases) in the disintegration of food. The major differences among the methods are the forms of enzymes (pancreatin or individual enzymes) and biliary acids used (bile salt mixtures, real fresh bile, or individual bile salts) [21]. 

The contribution of the intestinal step to the bioaccessibility of phenolic compounds is clearly influenced by several parameters. First, the action of intestinal enzymes on the residual matrix could increase the phenolic content. Next, polyphenols are chemically reactive in near-neutral conditions and their degradation or isomerization may be catalyzed by the presence of oxygen and/or transition metal ions. Moreover, specific absorption by the small intestine can occur by passive diffusion or active transport, as demonstrated for aglycones and their glucosylated forms. The latter forms can be actively transported by the sodium–glucose-linked transporter found in the enterocytes. Extracellular hydrolysis can be promoted by lactase phlorizin hydrolase in the brush border and be followed by diffusion of the resulting aglycone into the enterocyte [45]. Transcellular transport involving multidrug resistance protein and P-glycoprotein transporters appears to be favored for hydroxycinnamic acid and flavonol aglycones. These two phenomena cannot be readily modeled in vitro. Therefore, in vitro digestion methods may overestimate the levels of these phenolic components. Moreover, absorption is oversimplified but coupling of the dynamic digestion systems with cellular models (Caco-2, HT-29, IPEC-J2) could allow better simulation of epithelial transport. The absence of microbiota in the distal parts of the small intestine can appear as a limit [33]. 

Limiting oxygen levels, inclusion of α-glucosidase activities, sufficient bile salt concentration, and the presence of lipolytic, amylolytic and proteolytic enzymes for specific nutrient digestion are all of importance for an optimal release of phytochemicals. While remaining triglycerides may trap lipid-soluble phytochemicals, incompletely digested proteins and polysaccharides may bind to water-soluble phytochemicals, making them unavailable in the small intestine [21].

There are different ways of simulating the bioaccessible fraction of food at the intestinal level. The easiest approach is to analyze the resulting content of the entire intestinal fraction, just by its filtration to separate the soluble material (fraction available for uptake). In addition, dialysis and centrifugation are two common techniques that have also been used for simulating the bioaccessible fraction of food and extracts. In the dialysis model, the dialyzable fraction represents the sample that goes through the semi-permeable membrane and is available for absorption; meanwhile, the fraction outside the dialysis membrane represents the non-absorbable sample. In the solubility model, the intestinal sample is centrifuged to obtain a supernatant (soluble components that could be potentially absorbed) and a precipitate (unabsorbed compounds). Separation by centrifugation or filtration, followed by analysis of soluble components has been reported as a good estimate of compounds available for transport across the intestinal epithelium. In the case of dialysis, data should be carefully studied since parameters such as molecule dimensions, polymerisation degree and presence of sugar in the molecule, or even the membrane washing procedure may modify the amount of sample able to permeate through the membrane. However, when undigested compounds form colloidal dispersions, dialysis may be the better choice, since centrifugation will only separate the insoluble undigested material with sufficient density. Moreover, dialysis could be a useful tool for coupling the dialyzable fraction with cell lines without further purification steps [46].

### 4.4. Colonic Stage

The colon contains a complex microbial ecosystem, which can ferment food components not digested in the upper gastrointestinal tract. Some undigested food ingredients, e.g., certain polyphenols, may be substrates for the indigenous bacterial community [47]. Moreover, products from microbial bioconversion can affect the intestinal ecosystem and the bioavailability of the parent compounds. Characterization of phenolic metabolites using in vitro colonic models is complementary to the metabolic bioconversion by the small intestine or the liver and includes methylation, sulfation, and glucuronidation [48]. Colonic metabolism of phenolic compounds begins with the transient appearance of aglycones and the subsequent formation of hydroxylated aromatic compounds and phenolic acids. Flavones, flavanones, flavanols, proanthocyanidins, and phenolic acids share hydroxyphenylpropionic acid metabolites [49,50], while flavonols and ferulic acid dimers share hydroxylated phenylacetic acid metabolites [51]. Benzoic acid derivatives, hydroxylated benzaldehydes, and acetaldehydes are formed from anthocyanins [18]. Complex metabolites, such as lactones formed from plant lignans or ellagitannins are reabsorbed from the colon and are subject again to liver metabolism and the conjugate derivatives are excreted via urine [21]. Thus, plasma and urine excretions reflect both the hepatic and colonic metabolism of polyphenols.

## 5. Polyphenols: Structure–Antioxidant Activity Relationship 

Polyphenols are a well-known group of secondary plant metabolites that have been analyzed in detail and constitute a significant set of natural antioxidants that simultaneously reveal numerous biological activities [2,52]. The compounds principally arise from the shikimate synthesis pathway and phenylpropanoid metabolism. They play crucial roles in plant defense systems and are responsible for antibacterial, antifungal or antiviral activity. Additionally, the coloration and taste of many plant tissues result from their polyphenol content [53]. 

**Figure 1:**
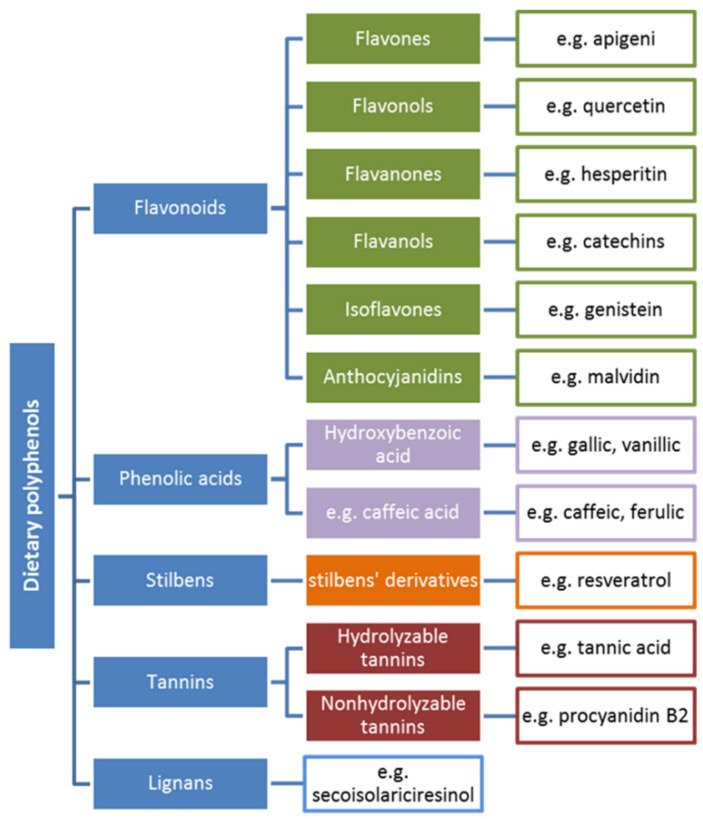
Dietary polyphenols—classification and examples [54].

On the whole, polyphenols can be divided into five structural groups that, in turn, consist of several subgroups. Polyphenols, along with examples, are presented in Figure 1. 

### 5.1. The Antioxidant Activity of Phenolic Acids and Flavonoids 

Numerous antioxidant studies have revealed the high activity of polyphenols on free radical scavenging ability. In accordance with Kumamoto et al. [55], the strong antioxidant activity results from the resonance stabilization of the polyphenolic radical that is obtained after oxidation processes as well as from the ability of the antioxidants to chelate transition metals (e.g., iron) [56]. Unfortunately, some conditions such as lipid system, high concentration of transition metal ions, alkali pH, presence of oxygen molecules, cause a pro-oxidant character of the compounds. This feature of polyphenols was extensively studied. The pro-oxidant character of some polyphenols results from the fact that small polyphenols are simply oxidized, whereas large-molecular-weight compounds are not so prone to pro-oxidant factors. Among the most oxidizable polyphenols are hydroxycinnamic acids (e.g., p-coumaric, rosmarinic, caffeic) which are able to damage DNA [56].

On the whole, polyphenols constitute a large group of compounds with one or more hydroxyl group linked to their aromatic rings. Polyphenols range from simple structures such as phenolic acids to complex forms such as tannins [57]. Similar to all other antioxidants, the structural form of the polyphenol decides its ability to scavenge free radicals. Of most importance is the degree of methoxylation and the number of -OH groups. Additionally, polyphenolic activity is closely connected with the ability to induce metal ion chelation via the o-dihydroxy phenolic structure in order to scavenge alkoxyl and peroxyl radicals as well as to regenerate *α*-tocopherol [57]. Below, the structure–antioxidant activity relationship of the most active groups of polyphenols is discussed.

#### 5.1.1. Phenolic Acids 

**Figure 2:**
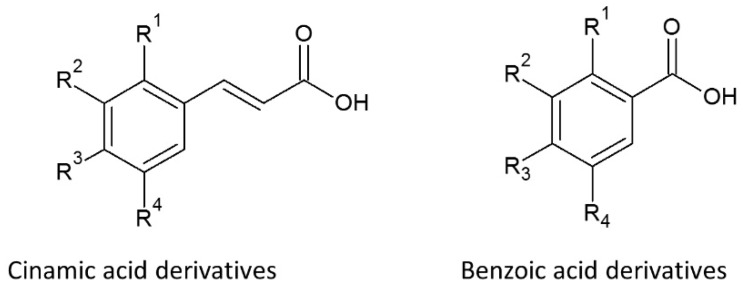
Structures of cinnamic and benzoic acid derivatives. Substitution of the hydroxyl group in the R position leads to the generation of the various phenolic acids. Examples of cinnamic acid derivatives include caffeic acid (R^3^ = R^4^ = OH), ferulic acid (R^2^ = OCH_3_, R^3^ = OH) and the benzoic acid derivatives: vanillic acid (R^2^ = OCH_3_, R^3^ = OH) and gallic acid (R^2^ = R^3^ = R^4^ = OH).

The structure of the phenolic acid compounds consists of a benzene ring and carboxyl and hydroxyl groups (Figure 2). The latter, along with steric effects, decide their antioxidant activity [58]. Antioxidant activity of the compounds is strictly related to the positioning of hydroxyl groups that are bound to the ring as well as the various types of substitution. Additionally, energy of the bond between the H atom and O atom in the hydroxyl group is less than in aliphatic compounds due to the -OH group isbonded to the aromatic ring system. This phenomenon results from the resonance effect of aromatic rings. The detachment of hydrogen from the -OH moiety during radical/antioxidant reactions leads to the creation of phenoxyl radicals wherein the relatively high stability results from the shift in charges in the ring. The reaction is followed by the creation of quinones or other reactions (e.g., dimerization), leading to stopping the free radical-antioxidant process [59,60]. 

Detailed studies have revealed that the free radical scavenging reaction is based on the Hydrogen Atom Transfer (HAT) mechanism. Additionally, it is known that *meta*-monohydroxy derivatives, in contrast to *ortho*- or *para*-positioning, display high antioxidant activity. Moreover, the activity increases in acids along with the presence of additional groups on the ring. The most active benzoic acid dihydroxy derivatives are compounds with the -OH moiety in the 3 and 5 positions, and the activity can be increased by substitution of an alkyl or methoxy group in the *ortho*- position by way of the -OH moiety. An example of this is gallic acid (-OH in the 3,4 and 5 positions), which has very high ability to scavenge free radicals [57,59]. 

#### 5.1.2. Flavonoids

In accordance with Figure 1, flavonoids constitute the most diversified group of polyphenols in terms of biological activity and they are the most widespread substances of plant origin [61]. The flavonoids consist of a 15-carbon atom (C6-C3-C6) benzoic ring and a phenylpropane unit. In the structure, a heterocyclic system containing oxygen can be observed. Hence, the compounds are considered as derivatives of benzo-*γ*-pyrone. In most cases, the structures include a double bond in the C-2 and C-3 positions and a carbonyl group in position C-4 [58,62]. Flavonoid structures contain numerous modifications that lead to different biological activity. In the case of flavonoids as antioxidants, their high free radical scavenging activity results from the following [58,63,64]:

A B-ring with an *ortho-*dihydroxy (catechol) group. This feature induces effective ROS and RNS scavenging ability, as well as the high stability of the created phenoxyl radical.

A C-ring with a 4-oxo group and a double bond between C-2 and C-3. This feature has influence on the dislocation of an electron in the B ring that brings about antioxidant activity.

A- and C-rings with 4-oxo groups and -OH groups near C-3 and C-5. These generate the maximum antioxidant activity.

**Figure 3:**
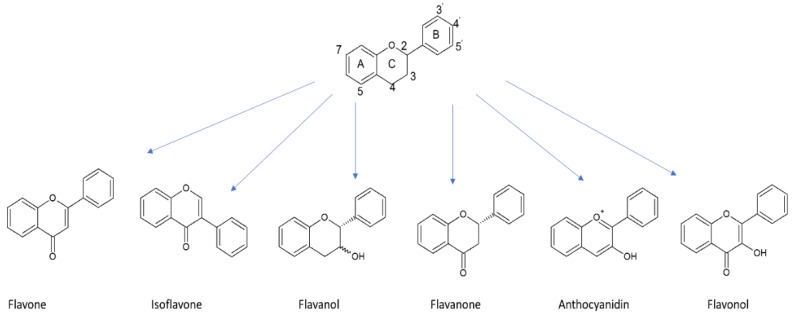
Classification of flavonoids.

Nevertheless, some changes in structure can reduce the high free radical scavenging ability. This effect can be observed in the case of glycosylation at the C-3 position or in the presence of a methoxyl group in the same position [65]. The basic structure of flavonoids and their subgroups are presented in Figure 3. 

As mentioned previously, in addition to their antioxidant activity, polyphenols can also act as pro-oxidants. This fact is explained by the metal-catalyzed oxidation of phenols leading to the formation of reduced transition metals. The new structures take part in the reduction in oxygen to peroxyl radicals and afterwards to hydroxyl radicals. Additionally, the metal ions can catalyze lipid oxidation. A positive side is the fact that polyphenols are able to scavenge peroxyl and hydroxyl radicals, as well as scavenge lipid-derived radicals [66].

### 5.2. Mechanisms of Free Radical Scavenging by Polyphenols and Influence of Reaction Conditions—General Information

Radicals can be scavenged by antioxidants by way of three mechanisms [67]: 

Hydrogen Atom Transfer (HAT), based on breaking the O–H bond:

ArOH + R^●^ → ArO^●^ + RH

Single Electron Transfer–Proton Transfer (SET-PT), where electron transfer is followed by proton release:

ArOH + R^●^ → ArOH^+●^ + R^−^ → ArO^●^ + RH

Sequential Proton Loss Electron Transfer (SPLET), when a proton is first lost:

ArOH → ArO^−^ + H^+^

ArO^−^ + R^●^ → ArO^●^ + R^●^

R^−^ + H^+^ → RH

The type of free radical scavenging mechanism activated depends on several factors such as antioxidant and free radical structure, as well as reaction environment (e.g., pH, solvents), etc. Herein, pH is a particularly important factor, with significant impact on antioxidant or pro-oxidant activity. This factor is often analyzed both in in vitro and in vivo studies, as free radical scavenger ability in physiological conditions depends on, aside from enzymes, various pH levels in different parts of the digestive tract. 

On the whole, in the case of phenols, higher pH causes an increase in their antioxidant activity. Simultaneously, this has a negative influence on the transition metals, leading to metal-catalyzed oxidation. Additionally, studies have revealed that low pH promotes the pro-oxidant activity of polyphenols in lipid dispersions [66]. The subject of pH influence can best be explained through examining a selected group of polyphenols diversified in terms of structure. The mechanism of action of the most active group of polyphenols and the influence of pH on the process are typified in the following Section (Section 5.2.1).

#### 5.2.1. Phenolic Acids 

The high free radical scavenging activity of phenolic acids depends on both the number and relative position of the OH groups that are linked with the aromatic ring. Study results suggest that the process can be based on three mechanisms: HAT, SET-PT and SPLET. All of the mechanisms lead to the formation of a corresponding phenoxyl radical that is more stable and less reactive than the free radical species. 

Studies have also revealed that the activity of hydroxybenzoic acids (HBAs) depends on the number of OH groups. The strength of this is as follows: monohydroxy < dihydroxy < trihydroxy. Particularly good scavenging activity has been observed for 3,4-DHBA and 2,3-DHBA [68]. Di Majo et al. [69] noted the positive influence of pH change on the antioxidant activity of phenolic acids. The results of his work indicate the particular significant influence of pH 3.5 and 7.4 on the activity of benzoic and cinnamic acids, namely, that both pHs enhanced the antioxidant activity of cinnamic acids (caffeic, sinapic, ferulic) over that of benzoic acids (gallic, syringic, vanilic). This was explained in that the proximity of the COOH substituent to the aromatic ring is not beneficial for the antioxidant activity of phenol acids, and the insertion of an ethylenic group between the phenyl ring and the caroboxyl group has a favorable effect on reducing the properties of the OH group. Additionally, the CH=CH-COOH group plays a role in stabilizing the radical by resonance [60,69]. 

**Figure 4:**
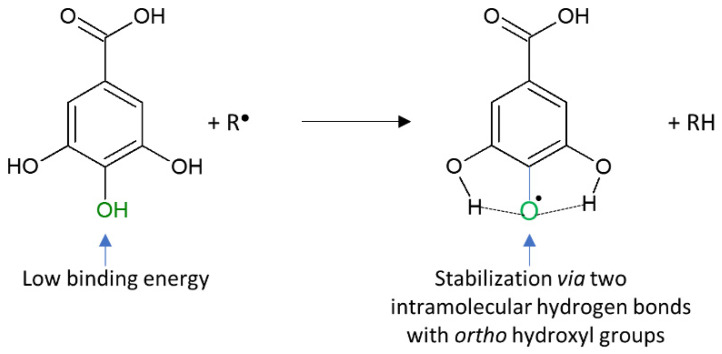
Gallic acid—free radical reaction and explanation of the stability of the obtained phenolic radical.

Figure 4 presents the gallic acid/free radical reaction and the factors responsible for the high antioxidant activity of the acid [70]. 

#### 5.2.2. Flavonoids

Flavonoids can act as highly active antioxidants by way of:

1. Metal chelation

**Figure 5:**
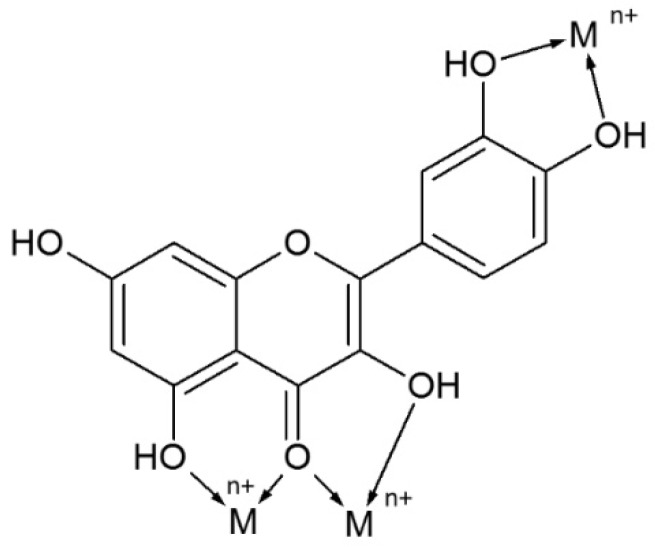
Metal chelation by quercetin—possible chelating sites.

It is known that flavonoids can chelate metals by more than one possible route. This depends on flavonoid structure, the type of metal ion and the pH of the reaction (e.g., in vivo conditions: acidic in the stomach and alkaline in intestine). In vitro studies have revealed numerous various dependencies between pH and the flavonoid moieties responsible for metal chelation. For example, in quercetin, the ortho-dihydroxyl group takes part in Fe^3+^, Cu^2+^ and Al^3+^ chelation in alkaline solutions, but can create complexes with Fe^3+^ (1:2) in acidic solutions with coordination via the 3–4 or 4–5 site and induce Fe^3+^ binding to the catechol group in a 1:1 metal/ligand ratio at higher pH [71,72]. Detailed analysis of all factors influencing flavonoid chelation ability was diligently undertaken by Kasprzak et al. [71]. Possible chelating sites of quercetin are presented in Figure 5.

2. Reduction in highly oxidizing free radicals

**Figure 6:**
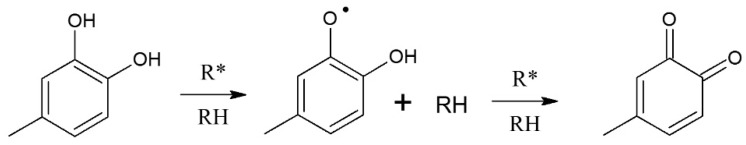
Flavonoids—free radical scavenging mechanism.

This activity results from the low redox potential of flavonoids, as this allows the generation of reducing radicals such as superoxide, peroxyl, alkoxyl and hydroxyl by hydrogen donation (Figure 6). The emerging aroxyl radical can then react with other radicals, leading to the formation of a stable quinone structure. The aroxyl radical can also react with oxygen, leading to the generation of quinone and superoxide anion rather than terminating the chain reaction. This situation can take place when the reaction environment is rich in transient metal ions [73]. In the case of the Fenton reaction, the most active flavonoids are compounds with 4-oxo units, catechol units and OH groups at the C-3 and C-5 position [58,74].

3. Inhibition of pro-oxidant enzymes

Flavonoids have revealed the ability to inhibit the enzymes (e.g., xanthine oxidase, protein kinase C) responsible for generating superoxide anions. Enzymes such as lipoxygenase, microsomal monooxygenase, and glutathione transferase can also be inhibited by the compounds [74,75]. 

Similar to other groups of polyphenols, the antioxidant activity of flavonoids is related to the pH condition of the reaction. Di Majo et al. [69] analyzed the influence of pH on the antiradical activity of flavonols and flavan-3-ols. The results revealed that kaempferol, quercetin and myricetin have the same antioxidant activity at pH 3.5, but that differences were observed at pH 7.4. At this pH, Kaempferol (one OH group in B ring) indicated lower activity than quercetin and myricetin (two and three OH groups in B ring, respectively). These differences can be explained by the fact that dihydroxy and trihydroxy structures are more active than kaempferol because the 3’,4’-catechol moiety is able to stabilize the corresponding radical through the formation of an intramolecular hydrogen bond [69]. Simultaneously, the positive influence of pH 7.4 can be explained by the fact that the antioxidant value of the compounds is a combination of the activity of the neutral as well as of the deprotonated form in different molar ratios, whereas, at pH 3.5, the neutral form is prevented [69]. This fact reveals that their antioxidant mechanism is based on hydrogen atom donation. Flavan-3-ols show the same trend as flavonols. The outcome (catechin activity > epicatechin activity) is due to the stereochemistry of the hydroxyl groups in the C-ring. Herein, the S-configuration of the OH group in the C-ring seems to be more favorable than the R-configuration. At physiological pH, the antioxidant activity of the two compounds can come about due to the combination of the activity of the neutral as well as the deprotonation form. Hence, the mechanism is based on hydrogen and/or electron donation. 

## 6. Parameters Affecting the Chemical Changes in Phytochemicals during Digestion

The bioaccessibility of polyphenols is influenced by many factors, such as the chemical state of the compound, the food matrix, interactions with other components or the presence of suppressors or cofactors, etc. Only substances released from the food matrix in the small and large intestine are digested. Phenolic compounds occur in foods mainly as esters, glycosides and polymers that cannot be absorbed in these native forms. They require hydrolysis by digestive system enzymes or intestinal microflora. It is estimated that 48% of all polyphenols are digested in the small intestine and 42% in the large intestine. Just 10% are undigested and remain intact within the food matrix. Only aglycones are able to pass through biological membranes on account of being highly lipophilic [29].

Among the most important factors determining bioavailability, and a prerequisite for intestinal absorption, is release from the food matrix and solubilization during digestion, also termed bioaccessibility [76]. In this manner, bioaccessibility is describing the fraction of a compound potentially available for further uptake and absorption. The amount of any bioaccessible compound may differ greatly from its total concentration in the food [27]. For this reason, understanding of the changes occurring during digestion is essential for the comprehension of bioaccessibility and for estimating bioavailability and bioactivity. For some phytohemicals that are poorly released and solubilized or that are degraded prior to reaching their site of absorption, the portion that is bioaccessible may be below 10% [30]. 

The digestion of phytochemicals is a complex process, and the bioaccessibility of compounds depends on the characteristics of the plant/food matrix, interaction of phytochemicals with other food components and the physiological conditions encountered in the various compartments of the gastrointestinal tract. Moreover, the physicochemical properties of the phytochemicals themselves are important parameters [21].

### 6.1. Impact of the Plant Matrix

An important factor influencing the bioavailability of polyphenols is the nature of the plant. Plant cell walls act as a barrier to digestion [77]. When a plant cell is broken (through mastication or crushing), phenolic compounds may associate with dietary fibers, leading to a modulation of their relative bioaccessibilities. Dietary fibers are the main carriers for phenolic compounds and thus influence their bioaccessibility, as fiber-entrapped polyphenols are both poorly extractable and barely soluble in the gastrointestinal fluids. High-molecular-weight proanthocyanidins and hydrolyzable tannins represent more than 75% of all food polyphenols ingested [78] and may bind tightly to dietary fibers, which restricts their accessibility. In wheat bran, ferulic acid and *para*-coumaric acid are mostly bound to arabinoxylans and lignin, and are thus insoluble, whereas sinapic acid is mainly found in soluble conjugate forms esterified to sugars and other compounds. Research has shown that the bioaccessibility of sinapic acid from bran-rich breads was much higher than that of ferulic acid and *para*-coumaric acid. Moreover, grinding of the bran fractions increased the bioaccessibility of phenolic acids [79]. Increased bioaccessibility was correlated to the presence of very small particles for sinapic acid and ferulic acid and to larger particles for *para*-coumaric acid. Of note, soluble and insoluble polysaccharides can bind phenolic compounds and limit their diffusion and substrate–enzyme contacts during gastrointestinal digestion, while increasing the viscosity of the medium [80].

### 6.2. Influence of Food Processing and Interaction of Phytochemicals with Other Food Components

The released number of phenolic compounds from the food matrix may be altered according to food composition, the way it is processed and the interaction of phytochemicals with other food components. Heat treatment may enhance polyphenol bioaccessibility due to disruption of plant tissue and denaturation of polyphenols–polysaccharide complexes. However, heat treatment may also cause thermal degradation of phenolic compounds [81]. Additionally, the interaction of phenolic compounds with other food components can modify their bioaccessibility. Studies have shown that the extractability of phenolic acids, flavonoids and proanthocyanidins appeared to be improved in the presence of fat, increasing by 1.5–3-fold for cocoa liquor (50% fat content) compared to cocoa powder (15% fat content) [82]. The affinity of milk and egg proteins, as well as gelatins for polyphenols depends on both protein and phenolic structures [83]. For example, chlorogenic acid associates with milk caseins rather than with *β*-lactoglobulin, and this complexation is relatively stable in simulated gastric and intestinal steps [84]. Furthermore, more than 60% of all green tea flavanols, which are very prone to oxidation, disappear in the intestinal phase during in vitro digestion [85]. A protective effect is induced by the addition of pure ascorbic acid, by citrus juices, as well as by bovine, rice and soy milks. While ascorbic acid contribution reflects its superior antioxidant capacity compared to tea flavanols, the protection by proteins is partially reversed by increasing the content of digestive enzymes, suggesting non-covalent interactions between bovine milk proteins and galloylated tea flavanols [86]. Soy isoflavones appear to be more bioaccessible from fruit juices and chocolate bars compared to cookies in in vitro conditions, perhaps due to their lower diffusion rate from the carbohydrate/ protein matrix of the cookies [87]. Similarly, the in vitro biaccessibility of catechin was significantly higher in beverages than in confections [88]. Higher amounts of isoflavones were also released in vitro from custards thickened with starch rather than with carboxymethylcellulose [89]. This effect is attributed to the hydrolysis of starch by α-amylase, which occurs from the mouth to the intestine.

## 7. Effects of Simulated Digestion on Phenolic Composition and Their Antioxidant Activity in Food 

### 7.1. Impact of Physiological Conditions Encountered in the Gastrointestinal Tract on Phenolic Composition

Due to the short interaction of oral enzymes with the food bolus prior to reaching the stomach, their influence is much less clear than the further digestion stages and rather limited to carbohydrate-rich foods [90]. However, Ginsburg and others [91] suggested that saliva plays an important role in the solubilization of polyphenols present in fruits and plant beverages. This substantially increases their availability. Moreover, saliva can boost adherence of polyphenols to oral surfaces and thus contributes to the enhancement of the redox status of the oral cavity. Salivary albumin, mucins and proline-rich proteins may be of particular importance, affecting the digestibility and absorption of specific polyphenols—for example, tannins may be precipitated by such proteins [92] through hydrogen bonding and hydrophobic interactions.

The hydrolysis of glycoside flavonoids already starts in the mouth by means of *β*-glycosidase action. However, its effectiveness is dependent on the types of sugars present in the molecule. Glucose conjugates are rapidly hydrolyzed, as opposed to others such as those of rhamnose [29]. Phenolic compounds can have strong affinities with human salivary proline- and histidine-rich proteins and form both non-covalent and covalent associations depending on the size of the phenolic compound [93]. High-molecular-weight polyphenols (such as tannins) can also interact strongly with fibers and proteins, but their affinity is related to their size and their solubility in water. More hydrophobic phenolic compounds bind more strongly to proteins [21,94]. 

In the stomach, where the pH is low, flavonoids oligomers degrade to smaller units. Of all the flavonoids, the flavon-3-ols exist as aglycones and pass in this form into the duodenum. In the small intestine, in the high pH, deglycosylation, glucuronidation, methylation, sulphonation and hydroxylation of flavonoids occurs. In these conditions, the flavonoid epigallocatechin gallate may become oxidized to more active forms for scavenging free radicals and chelating ionic iron [95]. In addition, in the stomach the absorption of free phenolic acids occurs as well as the phenolic acids can conjugate with glucuronic acid. Esters of phenolic acids are, however, degraded by the microbial esterases present in the large intestine. Undigested polyphenols then pass into the large intestine, where they are subjected to further degradation into phenolic acids by colonic microflora, as has been demonstrated in many studies [49]. In addition, glycosides are hydrolyzed by bacteria to aglycones that are then transformed into various acids through the action of *β*-glucosidase, *β*-rhamnosidase and esterases. Microflora enzymes can also catalyze the degradation of flavonoid chains into simple units. Moreover, they are able to perform hydrolysis, dehydroxylation, demethylation and decarboxylation. Depending on the structure of polyphenols, a large variety of compounds can be formed. Flavonols produce hydroxyphenylacetic acids, while flavones and flavanones degrade to hydroxyphenylpropionic acids. Furthermore, flavanols are degraded to both phenylvalerolactone and hydroxyphenylpropionic acids. Finally, metabolites of all these compounds lead to the generation of benzoic acid. They can be absorbed into the circulation, where they bind to albumin and are transported to the liver. Here, they undergo hydroxylation, demethylation, o-methylation, as well as conjugation to glucuronide and sulphated derivatives through phase I and II enzymes. A large portion of these can, in later stages, be secreted together with bile back into the gut where they again undergo hydrolysis and are either absorbed back or excreted via the feces [29]. 

Most phenolic compounds remain stable during salivary and gastric digestion. Gayoso et al. [46] evaluated the effect of different in vitro gastrointestinal digestion methods using three static models (filtration, centrifugation and dialysis) on the stability and bioaccessibility of phenolic compounds. Using absolute amounts of standards in the digested samples, rutin and caffeic acid showed recoveries of approximately 100% and rosmarinic acid approximately 85%–92% in all methods. However, when the results were referred to mg/mg lyophilized digested sample, the remaining % of sample decreased to 75% and 78% (oral and gastric, respectively) in the case of caffeic acid, and 67% and 68% (oral and gastric, respectively) for rosmarinic acid, maintaining the 100% in the case of rutin. Summarizing, no remarkable differences were observed between the initial amount subjected to digestion and the amounts recovered during the oral and gastric steps, showing that these processes hardly altered the stability of the three studied phenolic compounds. After the oral and gastric phases, a slight decrease in the antioxidant activity for caffeic acid and rosmarinic acid was observed (19%–12% and 36%–24%, respectively), whereas rutin showed no loss in the ability to scavenge free radicals [46]. A similar trend (loss in antioxidant capacity during intestinal digestion) was previously reported in the digestion of foods [96].

Bermúdez-Soto et al. [97] investigated the effects of in vitro gastric and pancreatic digestion (static model) on the stability and composition of the major polyphenols, including anthocyanins, in chokeberry juice. Herein, gastric digestion had no essential effect on any of the major polyphenols in samples. These results are in agreement with those reported for raspberry [98], pomegranate [99], bilberry and blackberry [100] anthocyanins. The high stability under the stomach conditions of flavonols or flavan-3-ols from chokeberry juice is also comparable to previous reported in vitro [101,102] and in vivo [98] stability. All the above findings are in agreement with previous studies, where compounds present in foods [96,103], plant extracts [104] and in pure phenolic compounds [105], such as phenolic acids and flavonols, demonstrated their stability under gastric conditions. Furthermore, it has also been described that acid pH during the gastric step protects polyphenols against degradation [106].

Recent studies have associated the low bioaccessibility of polyphenols with their interaction with dietary fiber, because it provides them with a physical barrier against the acidic gastric conditions, but their strong associations with cell walls avoid its absorption at the small intestine stage. However, this allows their retention in the non-digestible fraction, exhibiting further beneficial effects for their potential fermentation by human gut microbiota and the production of diverse metabolites with implications in human health [107].

The simulated digestion of anthocyanins from, for example, berries, red wine, and red cabbage, has shown that these compounds appear to be stable in the acidic conditions of the stomach, but less stable in the small intestinal pH [108,109]. The total recovery of anthocyanins from red cabbage was low (approximately 25%), possibly due to degradation into new phenolic components by the combination of the elevated pH and the presence of oxygen during pancreatic digestion [108]. In the large intestine, the metabolism of anthocyanins depends on splitting glycosidic bonds and breaking the heterocyclic anthocyanin chain, whereas metabolism of linseed lignans proceeds through microflora action, forming enterolactone and enterodiol products [29]. 

Another example is green tea flavanols, for which the stability order is epicatechin > epicatechin gallate > epigallocatechin = epigallocatechin gallate, which may reflect the higher oxidizability of the 1,2,3-trihydroxyphenyl moiety, as compared to that of 1,2-dihydroxyphenyl [29]. Anthocyanins, like most polyphenols, are largely affected by the alkaline conditions of pancreatic digestion. Herein, reported in vitro recoveries are between 70% and 20% [92,96]. For example, Bermúdez-Soto et al. [97] recovered approximately 57% of all cyanidin-3-glucoside after the in vitro pancreatic digestion of chokeberry juice. The differences may be attributed to factors such as the food matrix.

It has already been mentioned that polyphenols are highly sensitive to the mild alkaline conditions in the small intestine, where most dietary polyphenols are degraded or transformed into other compounds. Gayoso et al. [46] evaluated the bioaccessibility and antioxidant activity of rutin, caffeic acid and rosmarinic acid using three in vitro gastrointestinal digestion models: filtration, centrifugation and dialysis. At the intestinal level, a significant degradation of all compounds was observed when results were expressed on a concentration basis (mg/mg lyophilized sample), mainly due to the dilution effect that occurs during digestion. However, when results were expressed as absolute amounts (total mg in the digested fraction), this degradation was much lower, or even absent in the case of rutin. Moreover, bioaccessibility (in terms of total mg) was higher in filtration and centrifugation than in the dialysis method. A significant reduction in antioxidant activity was observed after the intestinal digestion of the three standards, regardless of the method used. Summarizing, it is difficult to compare bioaccessibility studies due to the many variables that may influence gastrointestinal digestion, such as the fraction used for their quantification and the units used for reporting the results. Therefore, the information obtained from the in vitro digestion processes should be carefully analyzed. The methodology and units used to report results are two critical parameters to take into account in bioaccessibility studies. 

Bermúdez-Soto et al. [97] presented results that are in line with the above studies. The authors carried out the pancreatic digestion of the polyphenols from chokeberry juice and discovered that these compounds were significantly altered during pancreatic action. This effect was more marked for anthocyanins (approximately 43% was lost during intestinal conditions), while flavonols and flavan-3-ols decreased by 26% and 19%, respectively. In addition, neochlorogenic acid decreased by 28%, whereas chlorogenic acid increased by 24%. Finally, interactions with the digestive enzymes were not found responsible for the observed losses that were mostly due to the chemical conditions during pancreatic digestion. These results show that dietary polyphenols are highly sensitive to the mild alkaline conditions in the small intestine. What is more, a significant portion of these compounds can be transformed into other unknown and/or undetected structural forms with different chemical properties and, consequently, different bioaccessibility, bioavailability and biological activity [97]. 

The sensitivity to autoxidation is probably overestimated in in vitro digestion models, as oxygen levels are lower in the gastrointestinal tract. Last, it should be noted that proteolytic enzymes could play a role in polyphenol bioaccessibility by releasing phenolic compounds bound to dietary proteins, as observed in the gastric tract for pepsin. However, more data support a role for phenolic compounds as inhibitors of intestinal enzymes such as trypsin and lipase [29].

Many studies point to higher polyphenol stability in the gastric phase and their degradation at the intestinal level (Table 2).

Other studies have, however, described significant losses of some phenolic compounds during salivary and gastric digestion [97,99,108,110,111,112,118,119]. Moreover, some studies also have reported high stability after the in vitro pancreatic digestion of compounds such as rosmarinic acid [120], pure quercetin and catechin [105], ellagic acid [110] or ferulic acid [118]. In general, the differences among studies may result from the effect of the food matrix and from the different experimental conditions applied. Pure compounds also showed high variability. For instance, the % of loss after intestinal digestion for rutin was found to be from only 3% [98] to total loss. In the case of chlorogenic acid, it was from 44% to 95.7% [119], and for quercetin, from 5.8% [105] to total loss [119]. Therefore, the digestion methodology seems to be a key factor for assessing bioaccessibility.

Characterization of phenolic metabolites using in vitro colonic models is complementary to the metabolic bioconversion by the small intestine or the liver (methylation, sulfation, and glucuronidation) of the native forms that are present in foods [21] and shows the diversity of structural transformations occurring in the colon prior to absorption. Colonic metabolism of phenolic compounds starts with the transient appearance of aglycones and the subsequent formation of hydroxylated aromatic compounds and phenolic acids. Flavones, flavanones, flavanols, proanthocyanidins and phenolic acids share hydroxyphenylpropionic acid metabolites [50], whereas flavonols (quercetin, myricetin) and ferulic acid dimers share hydroxylated phenylacetic acid metabolites [51]. Moreover, flavanols also yield hydroxyphenylvaleric acids and corresponding valerolactone derivatives [50]. Anthocyanins yield benzoic acids, hydroxylated benzaldehydes and acetaldehydes. Complex microbial metabolites, such as l ellagitannins, are reabsorbed from the colon and are subject again to liver metabolism, and the conjugate derivatives are excreted via urine [21].

### 7.2. Impact of Physiological Conditions Encountered in the Gastrointestinal Tract on the Antioxidant Activity of Polyphenols 

As mentioned previously, pH conditions have significant influence on both in vitro and in vivo studies. Researchers, thus, have considered the impact of this factor on the free radical scavenging mechanism of various classes of secondary metabolites. In many cases, the antioxidant activity is significantly different in acidic/alkaline environments in comparison to neutral environments. This aspect is very important for the theoretical consideration of the influence of the pH of selected parts of the digestive tract on the structures and activity of secondary plant metabolites that are supplied via food intake. Despite this aspect being very important for antioxidant consideration, available study results are limited. Research has revealed that the antioxidant activity of each extract is correlated with the number of OH groups in the main components, as well as their hydrogen-donating abilities. Furthermore, additional OH groups in *ortho*-positions have a positive influence on an increase in antioxidant activity, especially at pH 4. Thus, in order to explain the pH influence on extract activity, each component must be taken into account [121].

A vegetable rich in polyphenols is lettuce (*L. sativa*). Here, the antioxidant activity of an extract was determined at pH 4–9 [116]. *L. sativa* extract is rich in polyphenols such as chlorogenic acids, derivatives of caffeic acid and flavonoids [122]. The obtained results revealed that free radical scavenging ability increases with increasing pH, while pH 7 causes a slight decrease in the activity. The authors explained their observations by stating that the higher activity of polyphenyloxidase (PPO) due to pH is a determining factor in the expression of enzymatic activity. It can be said that catalysis of phenolics (e.g. chlorogenic acid) oxidation by PPO is stronger in higher pH. Moreover, pH-dependent increase in the antioxidant activity of phenols is due to an increase in their electron-donating ability upon deprotonation and their stabilization in alkaline solution, leading to polymerization reaction. The reaction can lead to the formation new oxidizable OH moieties in their polymeric products, resulting in a higher antioxidant activity [116].

Different results were obtained for sweet potato leaf extract (SPLE) [20]. Similar to the lettuce extract, SPLE is rich in chlorogenic acids and caffeic acids. In this case, a slightly alkaline (pH 8) environment had a negative influence on antioxidant activity, whereas neutral and weak acidic environments caused an increase in the activity. The differences can result from other components of the extracts such as the enzymes or secondary metabolites that influence polyphenol activity in the selected pH conditions.

Equally interesting studies were performed for grape marc extract [121]. The main identified polyphenols were gallic acid, procyanidins B1 and B2, polydatin, catechin, epicatechin, hydroperoxide, ferulic, chlorogenic and salicylic acids. The free radical scavenging ability was considered under the following pHs: 2.6; 3.7; 5.5; 7.4 and 8.0. Despite similar polyphenolic content to previous analyzed extracts, in this case, the pH value did not have significant influence on antioxidant activity. The highest activity was observed for pH 3.7, but it was not significantly different from initial pH (4.4.). However, statistically significant differences were observed for pH 3.7 and 2.6, as well as 3.7 and 5.5. 

The influence of pH on extracts of *Punica granatum, Ipomoea batatas L., Beta vulgaris, Daucus carota, Amaranthus paniculatus* and *Peucedanum graveolens* was also investigated [123]. The studies were based on three pHs: 4, 7 and 9. Here, pomegranate leaf extracts revealed higher antioxidant activity at all three pHs compared to the others. This outcome can result from the higher pH stability of phenolic compound components of the extract. Acidic pH turned out to be positive for carrot leaves, kilkeerae and pomegranate leaves for which pH 4 caused high antioxidant activity. On the other hand, alkaline pH was beneficial for sweet potato leaves and shepu for which antioxidant activity was the highest. 

Interesting studies were performed on the antioxidant activity of saffron honey [124]. Analysis of honey samples revealed that the substance is rich in flavonoids (hesperitin, apigenin, quercetin, luteolin) and phenolic acids such as caffeic, ellagic, chlorogenic, cinnamic, benzoic, vanillic and coumaric. Changes in antioxidant activity were observed under different pHs, from 3 to 6, and the researchers observed significantly decreasing activity with increasing pH. 

Phenolic compounds are mainly found in glycosylated, esterified or polymerized forms. Thus, during gastrointestinal digestion, they can be hydrolyzed as a consequence of the acid environment of the stomach, the alkaline environment of the intestine, and by the action of digestive enzymes [34]. These conditions result in several changes in the structure of these compounds, such as hydroxylation, methylation, dimerization and glycosylation, as well as in the formation of phenolic derivatives by the partial degradation of their original structure, as in the case of anthocyanins. Thus, the bioaccessibility of these compounds is highly dependent on their type and amount in the plant matrix. According to Castañeda-Ovando et al. [125], the antioxidant activity of phenolic compounds, mainly anthocyanins, is dependent on the pH of the medium. In addition, anthocyanins may present different structural conformations in different pH, and, therefore, they have higher or lower antioxidant capacity as new structures are formed. Sui et al. [126] showed that the increase in antioxidant capacity of anthocyanin-containing solutions was directly proportional to the increase in pH, as it varied from 2.2 to 6.0. Ribeiro et al. [115] found that in vitro gastrointestinal digestion significantly reduced the antioxidant potential of a juçara-based smoothie in both evaluated steps, namely gastric digestion and intestinal digestion. Accordingly, all the digested fractions revealing antioxidant potential, were always higher in the gastric digest. Although a higher concentration of phenolic compounds was observed in the intestinal digest of smoothie samples, the alkaline pH of the intestine reduced their antioxidant potential, as previously mentioned. In spite of this result, potential antioxidant compounds (ferulic, ellagic, vanillic and cinnamic acids) were also detected in the intestinal digest. Rodríguez-Roque et al. [96] submitted a soy drink rich in phenolic compounds to in vitro gastrointestinal digestion and observed a higher antioxidant capacity in the gastric digest as compared to intestinal digest. Thus, the reduction in antioxidant capacity under intestinal conditions can be attributed to the structural reorganization of some compounds due to their sensitivity to alkaline pH. In addition, these compounds are capable of binding to other constituents of the food matrix, resulting in the formation of complexes that may also contribute to the reduction in their antioxidant potential. 

## 8. Conclusions 

The in vitro gastrointestinal digestion of foods significantly influences the bioaccessibility of bioactive compounds such as phenolics. Since plant foods are often diverse in composition or eaten in conjunction with other foods, food bolus constituents can modulate the bioaccessibility and stability of phytochemicals. Therefore, defining the conditions that influence their absorption can provide significant insights into methods for maximizing the utilization of these potential health-promoting constituents. When considering in vitro bioaccessibility studies, chemical and biochemical reactions or physical constraints occurring within food must be taken into account. Factors in the bioaccessibility of polyphenols include their release from the food matrix, particle size, their hydrophilic/lipophilic balance as related to their glycosylation, different pH-dependent transformations (degradation, epimerization, hydrolysis, and oxidation within the gastrointestinal tract), and also interactions between polyphenols and food components. Knowledge about the breakdown of food constituents during digestion is very important because the possible effectiveness of plant metabolites for human health is mainly determined by the bioavailability of these molecules.

The present paper reviews some of the main in vitro digestion systems currently available. In vitro digestion protocols are widely used to address questions in the field of nutritional research. They are cheaper, faster and simpler to perform than in vivo experiments. Static models provide an inexpensive means to assess multiple experimental conditions, allowing large numbers of samples to be tested. Dynamic multistage continuous models facilitate long-term studies and come closest to in vivo conditions. All the systems presented in this review are not at the same stage of development. The TIM system was developed more than 20 years ago and has been regularly improved during all these years, while HGS or DIDGI^®^ were developed more recently. Dynamic in vitro digestion systems, when programmed with physiologically-relevant parameters, can mimic the complexity of the digestive process. However, when a system is validated for the digestion of a certain food, whether it is relevant for other types of foods needs to be researched, and it might be useful to validate those systems for, at least, families of foods with similar rheological properties. Other improvements could be envisaged to make these systems even more relevant. Absorption is oversimplified but coupling of the dynamic digestion systems with cellular models could allow better simulation of epithelial transport. In the future, dynamic digestion systems will probably become compulsory for understanding the mechanisms of food digestion, especially because of the increased ethical and economic constraints of in vivo trials.

## Figures and Tables

**Table 1:** Analysis of processes in the digestive tract.

Part of the Digestive Tract	pH	Substrates (Nutrient)	Enzymes	Digestion Products	References
**Mouth**	neutral	Starch, fats	salivary amylase (ptyalin), lingual lipase	maltose and dextrins, non-esterified fatty acids	[9,10,11]
**Esophagus**	neutral	moving food to stomach after initial enzymatic and mechanistic processes in mouth			[12]
**Stomach**	1.5–2.0	Peptides, emulsified lipids casein	Pepsin, lipase rennet	amino acids, glycerol, fatty acids, glycerides, curdle casein	[12,13]
**Small Intestine**	light alkaline, approx. 8	Polypeptides, starch sucrose, fats, proteins, starch/glycogen	Aminopeptidase, amylase, sucrose, lipase, chymotrypsin, pancreatic amylase	amino acids, maltose and dextrins, glucose and fructose, glycerol and fatty acids, amino acids, maltose and isomaltose	[13,14,15,16]
**Large Intestine**	neutral	absorption of water and salts, production and absorption of vitamins, propelling feces for elimination from organism			[17,18]

**Table 2:** In vitro gastric and intestinal simulated digestion of polyphenols.

Product	Phenolic Compounds	In Vitro Gastric Conditions	Results	In vitro Intestinal Conditions	Results	References
**Mango by-Product Snacks**	gallic acid, magniferin	pepsin, HCl, pH 1.5, 2 h	Small increase in polyphenols	pancreatin, buffer, pH 7.5, 6 h	90%–95% decrease in gallic acid, 95%–98% decrease in mangiferin	[109]
**Orange Juice**	flavanones	pepsin, HCl, pH 2.0, 2 h	No changes	pancreatin, bile, NaHCO_3_, pH 7.5, 2 h	50%–60% conversion into chalcones	[110]
**Pomegranate Juice**	anthocyanins	pepsin, HCl, pH 2.0, 2 h	10% increase	pancreatin, bile, NaHCO_3_, pH 7.5, 2 h	approximately 80% decrease	[99]
**Coffee Blend (65% Roasted, 35% Green)**	monohydroxy-cinnamoylquinic acids, dihydroxycinnamoyl-quinic acids, lactones, caffeoylshikimic acids, cinnamoyl amino acids	pepsin, HCl, pH 2.0, 2 h	recovery of the initial amount: monohydroxy-cinnamoylquinic acids 97%, dihydroxycinnamoyl-quinic acids 101%, lactones 39%, caffeoylshikimic acids 80%, cinnamoyl amino acids 74%	pancreatin, Britton-Robinson buffer, pH 7.5, 2 h	recovery of the initial amount: monohydroxy-cinnamoylquinic acids 67%, dihydroxycinnamoyl-quinic acids 108%, lactones 36%, caffeoylshikimic acids 55%, cinnamoyl amino acids 63%	[111]
**Broccoli**	flavonoids, hydroxycinnamoyl derivatives	pepsin, HCl, pH 2.0, 2 h	flavonoids stable, 6%–25% losses of cinnamics	pancreatin–bile, NaHCO_3_, pH 7.5, 2 h	approximately 80%–85% losses	[112]
**Apple Pomace**	flavanols, phenolic acids dihydrochalones flavonoids	pepsin, HCl, pH 2.0, 30 min	marked increase in flavanols, phenolic acids and dihydrochalones, no changes/small changes in flavonoids	pancreatin, buffer, pH 6.0, N_2_, 5 h	significant degradation of epicatechin, procyanidin, quercetin-3-o-galactoside, chlorogenic acid, phloridzin	[113]
**Soy Bread**	isoflavonoids	pepsin, HCl, pH 2.0, 1 h, N_2_	no changes	pancreatin, bile, NaHCO_3_, pH 6.9, N_2_, 2 h	isoflavonoids mostly stable; some conversion to aglycones	[114]
**Juçara-Based Smoothie**	anthocyanins, total polyphenols (TPC)	pepsin, HCl, pH 3.0, 2 h	the bioaccessibility of the anthocyanins was approximately 25%, the bioaccessibility of TPC was approximately 20%	pancreatin, bile, NaHCO_3_, pH 7.0, 2 h	the bioaccessibility of the anthocyanins was in the range of 7%–12%, the bioaccessibility of (TPC) was in the range of 40%–47%	[115]
**Raspberry**	anthocyanins	pepsin, HCl, pH 2.0, 2 h	no changes	pancreatin, bile, NaHCO_3_, pH 7.5, 2 h	30% losses of anthocyanins	[98]
**Onions, Apples**	quercetin, quercetin-3-glucoside	pepsin, HCl, pH 2.0, 30 min	no changes	pancreatin, bile, NaHCO_3_, pH 6.5, 1 h	50%–75% loss of quercetin, 10% loss of quercetin-3-glucoside	[101]
**Bamboo Leaves Soup**	total polyphenols (TPC)	pepsin, HCl, pH 2.0, 1 h	TPC increased by 1.64%	pancreatin, bile, NaHCO_3_, pH 7.4, 2 h	TPC decreased by 19.97%	[116]
**Yerba Mate**	caffeoyl glycosides, monohydroxy-cinnamoylquinic acids, dihydroxycinnamoyl-quinic acids, lactones, flavonoids	pepsin, HCl, pH 2.0, 2 h	recovery of the initial amount: caffeoyl glycosides 92%, monohydroxy-cinnamoylquinic acids 93%, dihydroxycinnamoyl-quinic acids 92%, lactones 99%, flavonoids 97%	pancreatin, Britton-Robinson buffer, pH 7.5, 2 h	recovery of the initial amount: caffeoyl glycosides 57%, monohydroxycinna-moylquinic acids 58%, dihydroxycinnamoyl-quinic acids 48%, lactones 45%, flavonoids 54%	[117]
